# Supplementary material for: Quantitative Proteomics Analysis of ABA- and GA3-Treated Malbec Berries Reveals Insights into H2O2 Scavenging and Anthocyanin Dynamics
Source: Plants (Basel). 2024 Aug 25;13(17):2366. doi: 10.3390/plants13172366 (PMC11396855; doi:10.3390/plants13172366)
Supplement: Supplementary file 1 [file plants-13-02366-s001.zip › Supplementary Figures and Tables.pdf]

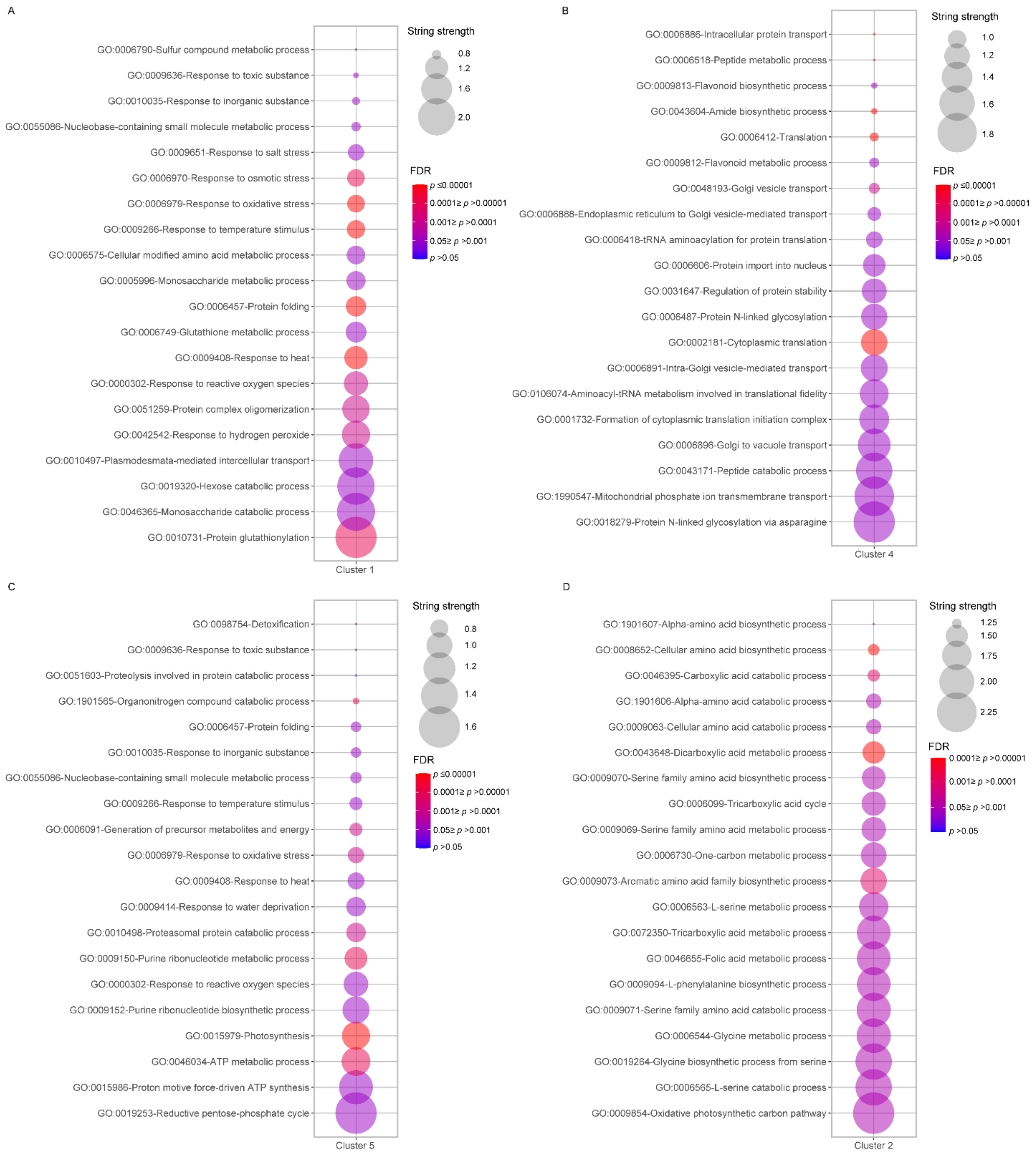

Supplementary Figure S1. Enrichment analysis of berry skins differentially abundant proteins (DAPs) at almost ripe stage (AR). The color gradient represents adjusted *p* values and the differences in bubble size correlate with the string strength [Log_{10}(Observed proteins in the network /expected proteins in a random network of the same size)]. GO: Gene ontology term for biological processes retrieved by STRING enrichment.

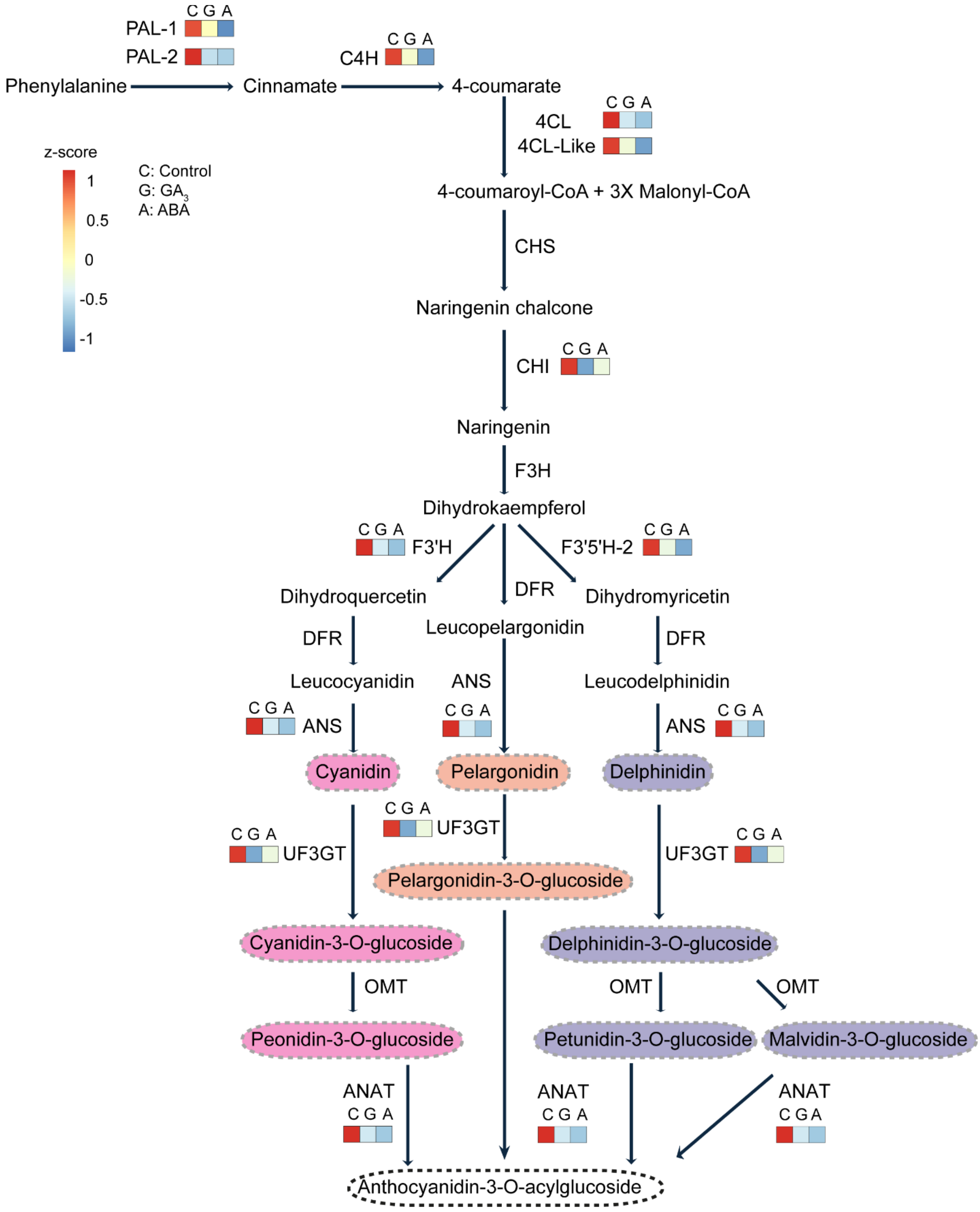

Supplementary Figure S2. Anthocyanins biosynthesis pathway. PAL-1: phenylalanine ammonia lyase-1 (F6HNF5), PAL-2: phenylalanine ammonia lyase-2 (A5BPT8), C4H: trans-cinnamate 4-monooxygenase (A5BRL4), 4CL: 4-coumarate-CoA ligase (F6GXF5), 4CL-Like: 4-coumarate-CoA ligase-Like (F6GW98), CHI:

chalcone-flavonone isomerase (F6HC36), F3'H: flavonoid 3'-monooxygenase (D7SI22), F3'5'H-2: flavonoid 3',5'-hydroxylase-2 (F6HA82), ANS: anthocyanidin synthase (A2ICC9), UF3GT: UDP-glucose flavonoid 3-O-glucosyltransferase (D7SQ45) and ANAT: anthocyanin acyltransferase (D7TU67). The values > 0 in the image indicate up-regulated, while the values < 0 indicate down-regulated.

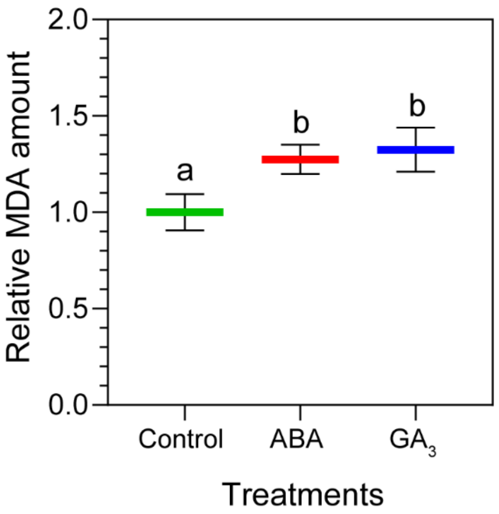

Supplementary Figure S3. Relative malondialdehyde (MDA) amount in control, ABA- and GA_3-treated berries at almost ripe stage (AR). Values are means ± SE, n = 3. One-way ANOVA followed by Fisher’s LSD test was applied. Different letters indicate significant differences (p< 0.05).

Supplementary Table S2. Anthocyanins predictive model using a linear mixed-effects regression. Fixed effects: TSS, total soluble solids (g $berry^{-1}$) and H_2O_2, hydrogen peroxide (nmol $berry^{-1}$). Random effect: Treatment (control, ABA and GA_3). Marginal R^2 (R^2m): represents the variance explained by the fixed effects; conditional R^2 (R^2c): represents the variance explained by the entire model. Significance codes: (***) *p*< 0.001, (**) *p*< 0.01, (*) *p*< 0.05.

**Fixed effects**	**Estimated**	**t-value**	***p*-value**	**Random effects**		**Variance**	**R^2**	
				**Groups**	**Name**		**R^2m**	**R^2c**
Intercept	-0.564 ± 0.142	-3.977	0.00102**		Intercept	0.005 ± 0.072		
TSS	5.280 ± 0.159	33.011	1.83e-10***	Treatment	TSS	0.013 ± 0.115	0.95	0.98
H_2O_2	0.001 ± 0.0004	2.418	0.03490*		H_2O_2	8.52e-08 ± 0.0003		
				Residual	---	0.010 ± 0.102		
